# QTL mapping of shoot and seed traits impacted by Drought in Barley using a recombinant inbred line Population

**DOI:** 10.1186/s12870-023-04292-x

**Published:** 2023-05-27

**Authors:** Oyeyemi O. Ajayi, Phil Bregitzer, Kathy Klos, Gongshe Hu, Jason G. Walling, Ramamurthy Mahalingam

**Affiliations:** 1 Cereal Crops Research Unit, USDA-ARS, 502 Walnut Street, Madison, WI 53762 USA; 2 Small Grains and Potato Germplasm Research, USDA-ARS, Aberdeen, ID USA

**Keywords:** Barley, Drought, Golden promise, Malting, Otis, Quantitative trait loci mapping, Recombinant inbred lines

## Abstract

**Background:**

With ongoing climate change, drought events are severely limiting barley production worldwide and pose a significant risk to the malting, brewing and food industry. The genetic diversity inherent in the barley germplasm offers an important resource to develop stress resiliency. The purpose of this study was to identify novel, stable, and adaptive Quantitative Trait Loci (QTL), and candidate genes associated with drought tolerance. A recombinant inbred line (RIL) population (n = 192) developed from a cross between the drought tolerant ‘Otis’ barley variety, and susceptible ‘Golden Promise’(GP) was subjected to short-term progressive drought during heading in the biotron. This population was also evaluated under irrigated and rainfed conditions in the field for yields and seed protein content.

**Results:**

Barley 50k iSelect SNP Array was used to genotype the RIL population to elucidate drought-adaptive QTL. Twenty-three QTL (eleven for seed weight, eight for shoot dry weight and four for protein content) were identified across several barley chromosomes. QTL analysis identified genomic regions on chromosome 2 and 5 H that appear to be stable across both environments and accounted for nearly 60% variation in shoot weight and 17.6% variation in seed protein content. QTL at approximately 29 Mbp on chromosome 2 H and 488 Mbp on chromosome 5 H are in very close proximity to ascorbate peroxidase (*APX*) and in the coding sequence of the Dirigent (*DIR*) gene, respectively. Both *APX* and *DIR* are well-known key players in abiotic stress tolerance in several plants. In the quest to identify key recombinants with improved tolerance to drought (like Otis) and good malting profiles (like GP), five drought tolerant RILs were selected for malt quality analysis. The selected drought tolerant RILs exhibited one or more traits that were outside the realms of the suggested limits for acceptable commercial malting quality.

**Conclusions:**

The candidate genes can be used for marker assisted selection and/or genetic manipulation to develop barley cultivars with improved tolerance to drought. RILs with genetic network reshuffling necessary to generate drought tolerance of Otis and favorable malting quality attributes of GP may be realized by screening a larger population.

**Supplementary Information:**

The online version contains supplementary material available at 10.1186/s12870-023-04292-x.

## Introduction

Global warming and unpredictable climatic conditions have become a defining challenge of our times and a serious threat to farmers’ livelihood and global food supply. Drought affects production of food crops resulting in significant economic, social and ecological losses [1]. World population is expected to increase from 7.7 billion (currently) to 9.7 billion in 2050 [2], and this poses a significant risk to global food security. Abiotic stresses, especially drought, present a major challenge to sustainable food production, as they can reduce the potential yields by up to 70% in crop plants [3, 4]. Breeding plants possessing multiple adaptive strategies to overcome water stress has intensified in the past decade due to an increase in drought prone areas [5]. With world’s food demand expected to increase by more than 70% before 2050 [6], coupled with a projected greater than 50% reduction in grain production due to extreme drought [7], identifying germplasm resources with strong adaptation to drought-prone environments without compromise in yield and quality has become a top priority for commercial crop breeders.

Barley (*Hordeum vulgare* L.) is the fourth most important cereal crop globally [8], and is important for animal feed, malting, brewing, and food industries [9]. On average, approximately $1 billion is generated annually from feed barley and malt exports [10]. Although barley is well adapted to a wide range of climatic conditions [11], significant yield penalties (49–87%) has been reported [12]. Drought tolerance is a complex quantitative trait, controlled by many genes and numerous physiological mechanisms [13, 14]. Therefore, the evaluation of physiological responses in water limiting environments is an important step in determining the genetic basis of these traits and will be useful for developing drought tolerant cultivars.

To determine the genetic basis of complex traits, genetic, and genomic resources have been developed in a wide range of species [15–17], including barley [18, 19]. Quantitative trait loci (QTL) analysis has been an effective approach for linking genomic regions to target traits. Single nucleotide polymorphism (SNP) markers are sequence variations in eukaryotic genomes that are a useful resource for mapping QTL and for marker assisted selection (MAS) [20]. Morphological and physiological variations under drought stress provide the basis for selection of genotypes with high adaptability to drought stress [21]. A gamut of agronomic, morphological, physiological, and metabolic traits have been extensively used for screening for drought tolerance, including yield [22], leaf and root morphologies [23], biomass [24], leaf relative water content [25], accumulation of amino acids [25], stomatal conductance [26], photosynthetic parameters [27], and chlorophyll fluorescence [28]. These traits are correlated with drought response phenotypes and can be used as proxy for selection of drought tolerant genotypes in barley breeding programs [10, 29].

Several drought responsive QTL studies in barley employing recombinant inbred line (RIL) populations have been reported [30–34]. The population sizes in these studies ranged between 100 and 150 RILs and were either conducted under controlled greenhouse conditions [31, 35] or in the field [30, 33, 34]. Interestingly, three of the above studies were conducted using the same RIL population derived from Tadmor, a pure line selection from Arabi Aswad an extremely popular landrace from Syria and Er/Apm, a breeding line with high yield potential [36]. However, comparative analysis of the RILs that were consistently performing well under drought stress in the greenhouse and field studies was not considered nor was any seed quality traits evaluated. Secondly, the number of markers used for QTL mapping in the older studies and even the 2017 study [31] was less (817 markers) and hence detection of informative QTL may have been compromised.

Otis is a US spring feed barley variety with tolerance to abiotic stresses and Golden Promise is a spring malting variety that is sensitive to drough, heat and combined drought and heat stress [37]. In this study, 192 F_5:6_ RILs developed from a cross between Otis and Golden Promise, was genotyped using the 50k iSelect SNP Array (Illumina Inc., San Diego, CA, USA). The parental lines and the RILs were subjected to short term progressive drought stress in the biotron. The population also was evaluated in the field under irrigated and rainfed conditions. The objectives of this study were: (1) to identify stable QTLs responsive to water deficit; (2) to identify candidate genes underlying hotspots of QTL under water deficit conditions that may be useful in marker assisted selection in barley breeding programs focused on improving drought tolerance (3) to identify recombinant lines that exhibit drought tolerance in the different environments and analyze their malting quality attributes.

## Results

### Phenotypic and genetic variability of the mapping population

**Fig. 1:**
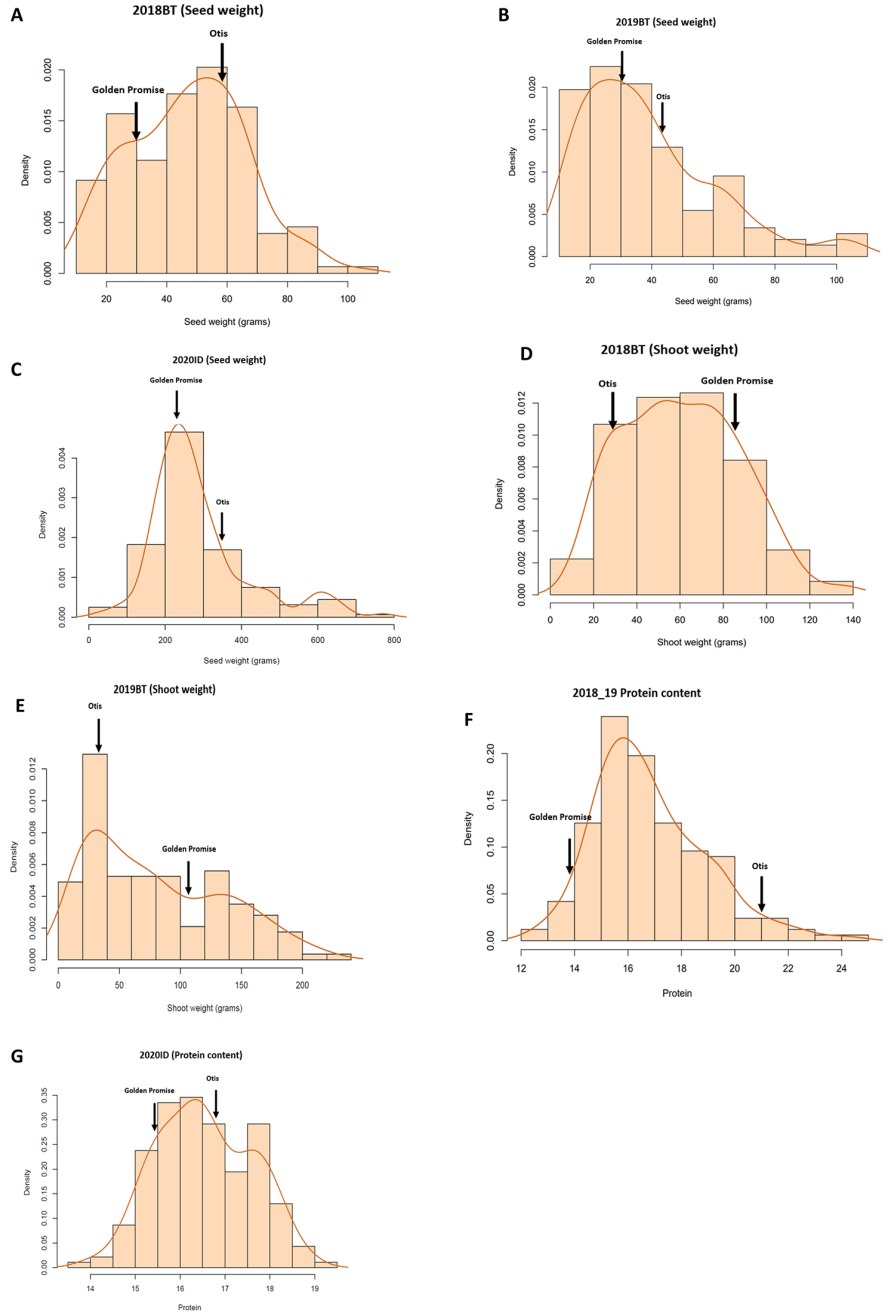
Frequency distribution of trait per environment in the GP X Otis recombinant inbred line (RIL) population. Values represented mean of each RIL. Parental values for each trait were indicated by arrows. X-axis represents the probability density estimation of the frequency of lines. Data obtained in each environment considered were indicated above each plot. For example, 2018BT and 2019BT represents biotron (BT) grown barley plants for year 2018 and 2019 respectively, while 2020ID represents field grown barley plants in Idaho for year 2020. Traits measured in those environments were indicated in brackets. **A-C** represents seed weight, **D** and **E** represents shoot dry weight, while **F** and **G** represents protein content

In both the controlled environment of the biotron, as well as in the field study, Otis was tolerant and GP was sensitive to water deficit based on seed yield per plant. It is important to note that in the biotron experiments the drop in the moisture level in the pots subjected to drought were significant within 48 h after with-holding irrigation. The moisture level in the pots containing the fully-grown plants were less than 20% for a period of 48–72 h, during the five days of water withholding used in this study. Otis showed higher seed yield and protein content than GP. GP had greater shoot dry weight than Otis in response to water deficit. For the RIL population, seed weight, shoot weight, and protein content measurements showed broad and continuous range of variation and the population average of each trait were close to the mid-parent values. The maximum and minimum values recorded for the RILs clearly showed transgressive segregation (Table 1). Seed weight, shoot weight and protein content measurements in different environments showed a normal distribution (Fig. 1A-D, F); although, bimodal distributions were observed for shoot weight in 2019 biotron (Fig. 1E) and 2020 protein measurements of Idaho field evaluation (Fig. 1G). Descriptive statistics including mean values and ranges for seed and shoot weight, and seed protein content of the mapping parents and the RIL population in both biotron and field are shown in Table 1.

**Table 1:** Variation in seed and shoot weight and protein content for Golden Promise, Otis and their RIL population

Trait^*a*^	Env^*b*^	Parents			RIL Population									
		P1	P2	P1-P2	Max	Min	Average	SD	Kurtosis	Skewness	CV	*H*		
SW ^*c*^	2018BT	32.32	60.98	-28.66	104.52	10.2	43.01	19.38	2.64	0.17	0.41	0.69		
	2019BT	31	44.07	-13.07	107.97	10.22	34.01	21.8	3.89	1.1	0.6			
	2020ID	243.16	340.57	-97.41	769.96	25.96	289.79	130.5	4.78	1.35	0.45			
ShW	2018BT	88.67	39	49.67	139	12	59.88	26.99	2.45	0.28	0.44	0.84		
	2019BT	114.71	36.2	78.51	222	11	76.73	54.93	2.23	0.6	0.68			
P	2018_19BT	13.95	21.04	-7.09	24.3	12.33	16.82	2.09	3.72	0.8	0.12	0.62		
	2020ID	15.52	16.78	-1.26	19.02	13.87	16.53	1.07	2.31	0.06	0.065			

^*a*^ SW, Seed weight; ShW, shoot weight; P, protein content of seeds. ^*b*^ 2018BT, year 2018 in biotron; 2019BT, year 2019 in biotron; 2018_19BT, samples from 2018BT and 2019BT combined; 2020ID, year 2020 in Idaho. ^*c*^ For the 2018BT and 2019BT the values represent average seed weight per plant and for the 2020ID it is the average seed weight per head row. P1, Golden Promise; P2, Otis, *H –* heritability

**Fig. 2:**
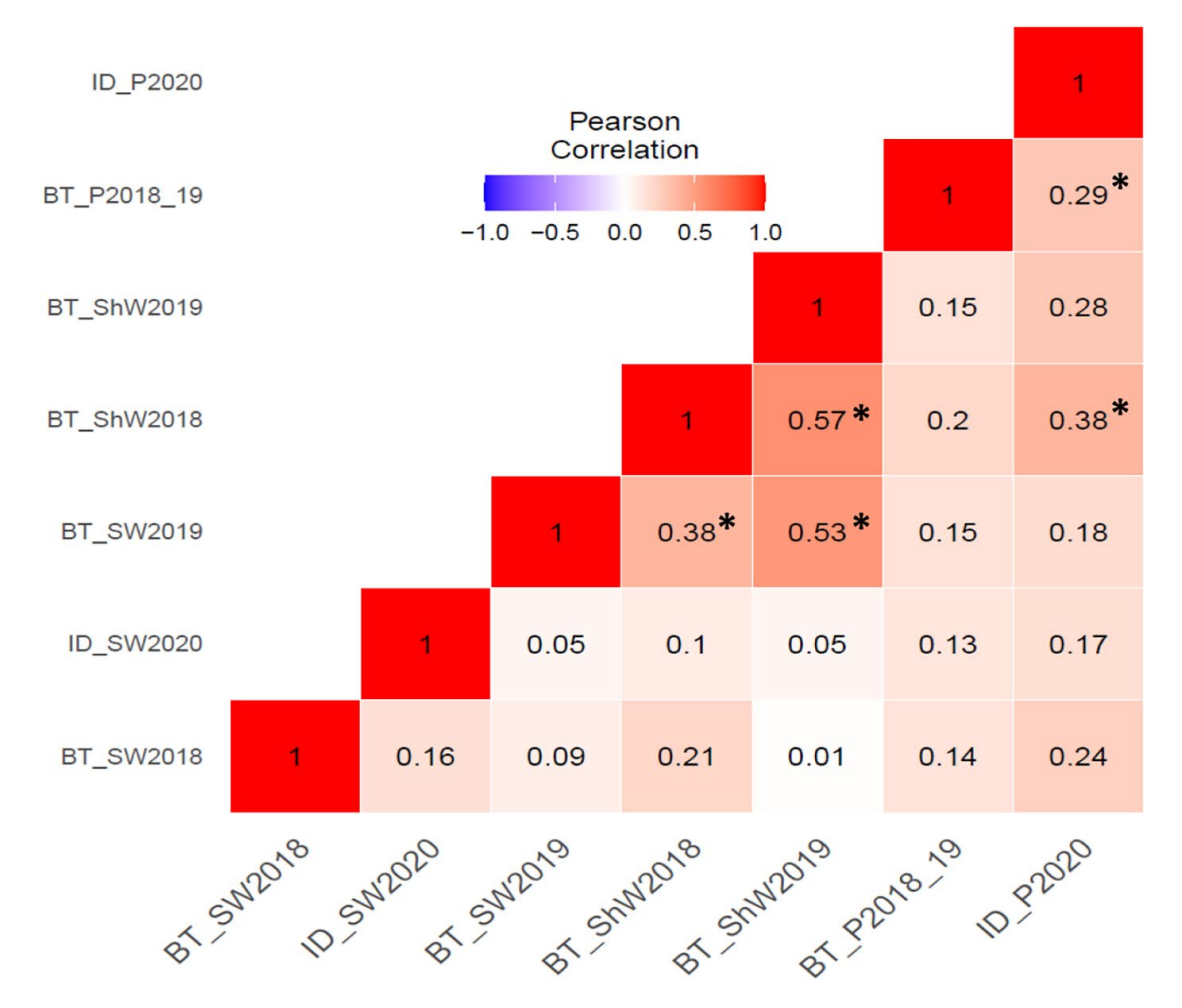
Heat map of the correlation matrix among traits based on mean values of each RIL within individual environment. Matrix was ordered by hierarchical clustering. Correlation between environments was not significant unless indicated by * (which means it is significant at P < 0.05 level). SW, Seed weight; ShW, shoot weight; P, NIR protein content of seeds; BT, biotron; ID, Idaho; 2018_19, samples from 2018BT and 2019BT combined.

Analysis of variance showed highly significant genotypic and environmental effects (P < 0.01) for all the traits investigated (Table 2). Broad sense heritability estimates were high for shoot weight (0.84) and moderate for seed weight (0.69) and protein content (0.62) (Table 1). Correlation analysis among traits and within individual environment was investigated (Fig. 2). While positive correlations were observed for traits within individual environments, significantly high positive correlations were mostly observed over the years for shoot weight and seed weight in the biotron.

**Table 2:** Analysis of variance (ANOVA) for seed weight, shoot weight, and protein content for the Golden Promise X Otis RIL population

Trait	Factor	DF^*b*^	Mean Square	F
SW^*a*^	Env	2	1170.36	591.09**
	Genotype	183	16.12	8.14**
	Error	314	1.98	
ShW^*c*^	Env	1	114.83	166.42**
	Genotype	182	1.56	2.26**
	Error	148	0.69	
P^*d*^	Env	1	6.87	7.56*
	Genotype	185	2.47	2.71*
	Error	165	0.91	

** Indicates significance at the 0.01 level. ^*a*^SW, seed weight; ShW, shoot weight; P, protein content of seeds. ^*b*^Missing data were excluded during ANOVA analysis. ^*c*^shoot weight data from biotron in year 2018 and year 2019. ^*d*^ protein content of pooled seeds from biotron 2018 and 2019, and field harvested seeds from Idaho from year 2020

### Genetic map construction

Between the two mapping parents (GP and Otis) 10, 810 SNP (21.6%) were polymorphic and scorable in the RIL population. After filtering out co-segregating markers, 4,617 SNP (9.23%) polymorphic markers were used for genetic map construction. Two RILs were identified as duplicates and were removed from this analysis. Average distance between mapped loci was about 1.0 cM and the genetic map spanned 4,566 cM distributed across seven chromosomes. Chromosomes 2 H, 5 H, and 7 H were enriched with more SNP loci compared to chromosomes 1 H, 3 H, 4 H, and 6 H.

### QTL and candidate gene analysis

**Fig. 3:**
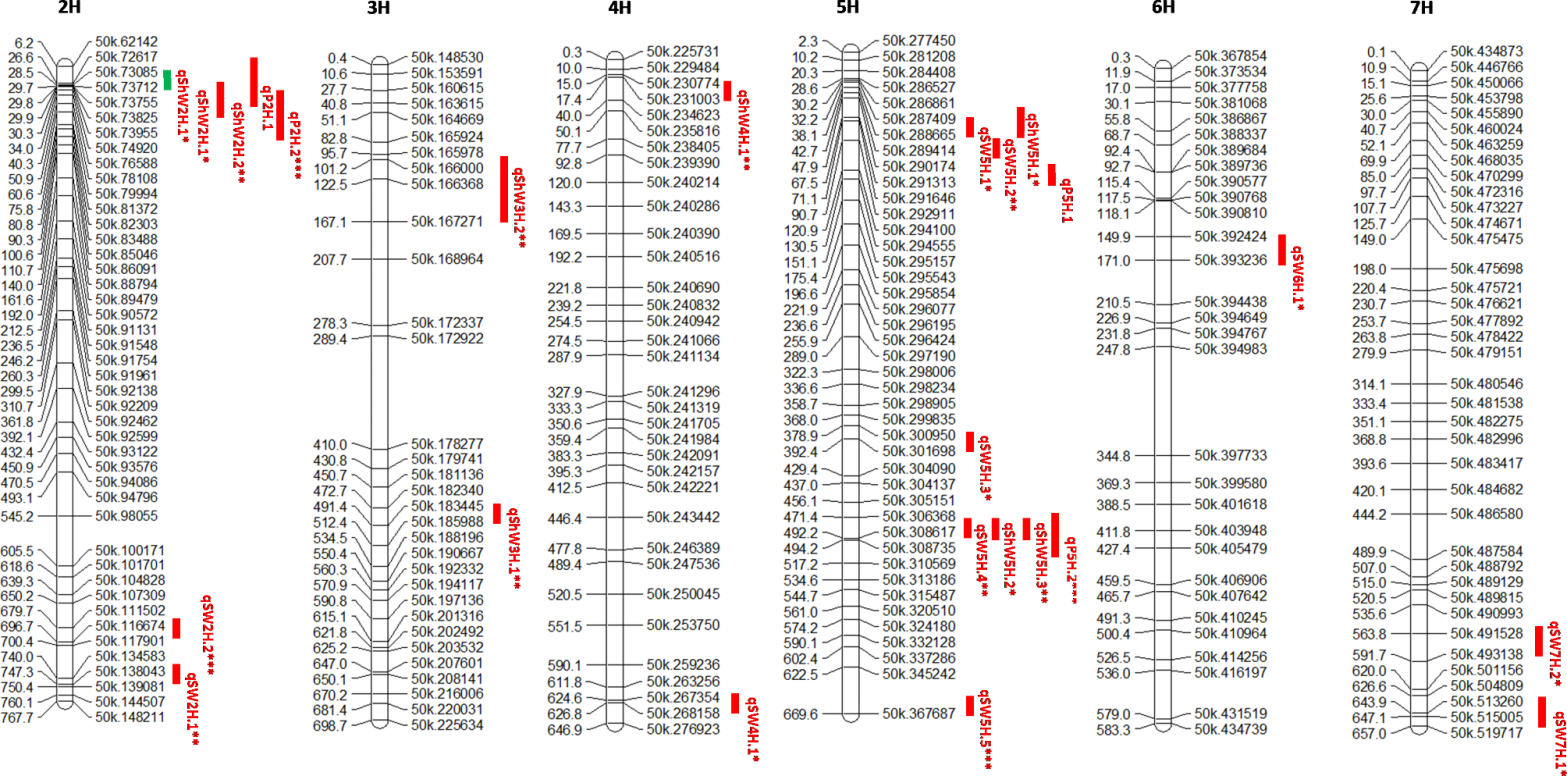
Chromosomal locations of QTL detected for seed and shoot weight and protein content in the GP X Otis recombinant inbred line (RIL) population in different environments. QTL intervals were indicated by thick red lines and represent one LOD score drop. *, **, *** corresponded to year 2018 in biotron, year 2019 in biotron and year 2020 in Idaho respectively. The thick green line on chromosome 2 H contained a qtl confidence interval region with LOD scores > 3 and close to the ascorbate peroxidase gene known to be involved in abiotic stress tolerance (See Fig. 4)

A total of eleven QTL were identified for seed weight distributed across chromosome 2 H, 4 H, 5 H, 6 and 7 H (Table 3; Fig. 3, Additional Table 2), explaining 3.12–14.11% of the total phenotypic variance with LOD values ranging from 2.11 to 3.13. Five of the QTL were on chromosome 5, two each on chromosomes 2 and 7, and one each on chromosomes 4 and 6. Otis, a thermotolerant cultivar contributed the ‘tolerant’ alleles and positive additive effects at 10 QTL loci to increase relevant trait values, whereas only one suggestive QTL (*qSW7H.2)* derived from GP influenced seed weight. The QTL *qSW2H.2* had the largest phenotypic variance explained accounting for 14.11% of the total variation influencing seed weight trait in field study. Overall, the variation explained by each significant QTL for seed weight from field experiment was higher than the biotron studies. Although majority of the QTL influencing seed weight was detected in a single environment and are in the telomeric regions, *qSW5H.1* and *qSW5H.2* were detected in the 2018 and 2019 biotron trials and co-localized in the same genomic region. Both QTL at this region explained little phenotypic variation in seed weight but with comparatively large effects compared to other QTL detected in biotron 2018 and 2019.

**Table 3:** Stable drought responsive QTL associated with seed weight, shoot weight and seed protein content in the Golden Promise X Otis RIL population

Trait^*a*^	QTL	Marker	Env ^*b*^	Flanking markers	Nearest locus	Marker position (bp)	LOD	Effect ^*c*^	PVE^*d*^ (%)
SW	**qSW5H.1**	JHI-Hv50k-2016-288072	2018BT	50k_287409-50k_288665	50k_288003	34,097,174	3.03	11.5	3.42
	**qSW5H.3**	JHI-Hv50k-2016-301278	2018BT	50k_300950–50k_301698	50k_301293	385,116,928	3.13	10.4	3.49
	qSW7H.2	JHI-Hv50k-2016-491735	2018BT	50k_491528–50k_493138	50k_491919	573,570,818	2.63	-10.3	6.54
	qSW2H.1	JHI-Hv50k-2016-138170	2019BT	50k_138043–50k_139081	50k_138043	747,544,069	2.11	7.2	5.02
	qSW5H.2	JHI-Hv50k-2016-288733	2019BT	50k_286527–50k_289414	50k_288700	38,516,658	2.13	11	3.66
	qSW5H.4	JHI-Hv50k-2016-308379	2019BT	50k_307471–50k_308766	50k_308374	487,888,257	2.49	11.2	3.12
	qSW2H.2	JHI-Hv50k-2016-116946	2020ID	50k_116674–50k_117901	50k_116867	697,001,696	2.27	15	14.11
	qSW4H.1	JHI-Hv50k-2016-267912	2020ID	50k_267354–50k_268158	50k_267984	626,279,933	2.34	52	12.97
	qSW5H.5	JHI-Hv50k-2016-367687	2020ID	50k_367687–50k_367854	50k_367317	669,579,327	2.37	5	6.88
	qSW6H.1	JHI-Hv50k-2016-390810	2020ID	50k_390577–50k_392424	50k_390577	118,108,028	2.77	24	12.53
	qSW7H.1	JHI-Hv50k-2016-513501	2020ID	50k_513260–50k_515005	50k_513580	644,608,283	2.14	51	10.98
ShW	**qShW2H.1**	JHI-Hv50k-2016-73562	2018BT	50k_73085–50k_73712	50k_73569	29,299,140	6.75	-21.9	24.13
	qShW5H.1	JHI-Hv50k-2016-287256	2018BT	50k_286861–50k_288665	50K_287256	31,678,885	2.87	14.8	6.91
	qShW5H.2	JHI-Hv50k-2016-308239	2018BT	50k_307515–50k_308584	50k_308332	487,200,167	2.66	14.1	7.47
	**qShW2H.2**	JHI-Hv50k-2016-73569	2019BT	50k_73085–50k_73951	50k_73566	29,301,310	4.78	-41.9	18.19
	**qShW3H.1**	JHI-Hv50k-2016-184654	2019BT	50k_183445–50k_185988	50k_184653	500,563,495	3.65	-36.2	10.25
	qShW3H.2	JHI-Hv50k-2016-167271	2019BT	50k_165978–50k_166368	50k_166000	167,105,550	2.94	-29.2	4.03
	qShW4H.1	JHI-Hv50k-2016-230796	2019BT	50k_230774–50k_231003	50k_230808	15,492,571	3	33.4	12.73
	**qShW5H.3**	JHI-Hv50k-2016-308419	2019BT	50k_307471–50k_308617	50k_308379	487,895,674	3.24	34.2	17.57
Protein	qP2H.1	JHI-Hv50k-2016-73085	2018_19BT	50k_72617–50k_73825	50k_73087	28,455,236	2.18	-1	4.68
	qP5H.1	JHI-Hv50k-2016-290678	2018_19BT	50k_290174–50k_291313	50k_290931	58,100,436	2.47	-0.9	5.28
	**qP2H.2**	JHI-Hv50k-2016-73825	2020ID	50k_73755–50k_74920	50k_73834	29,918,572	3.19	-0.6	6.63
	**qP5H.2**	JHI-Hv50k-2016-308379	2020ID	50k_306368–50k_308735	50k_308419	487,888,257	5.06	0.8	10.98

^*a*^SW, Seed weight; ShW, shoot weight; P, NIR protein content of seeds. ^*b*^2018BT, 2018 in biotron; 2019BT, 2019 in biotron; 2018_19BT, seed samples from 2018BT and 2019BT combined; 2020ID, 2020 in Idaho. ^*c*^Positive value indicated that Otis alleles increased the phenotypic value, negative value suggested that Golden Promise alleles increased the phenotypic value. ^*d*^ Phenotypic variance explained. QTL indicated in bold are with LOD scores above genome-wide significance threshold. QTL designations in normal font are suggestive QTL [77]. Marker positions (bp) were obtained from GrainGenes database (http://www.graingenes.org)

**Fig. 4:**
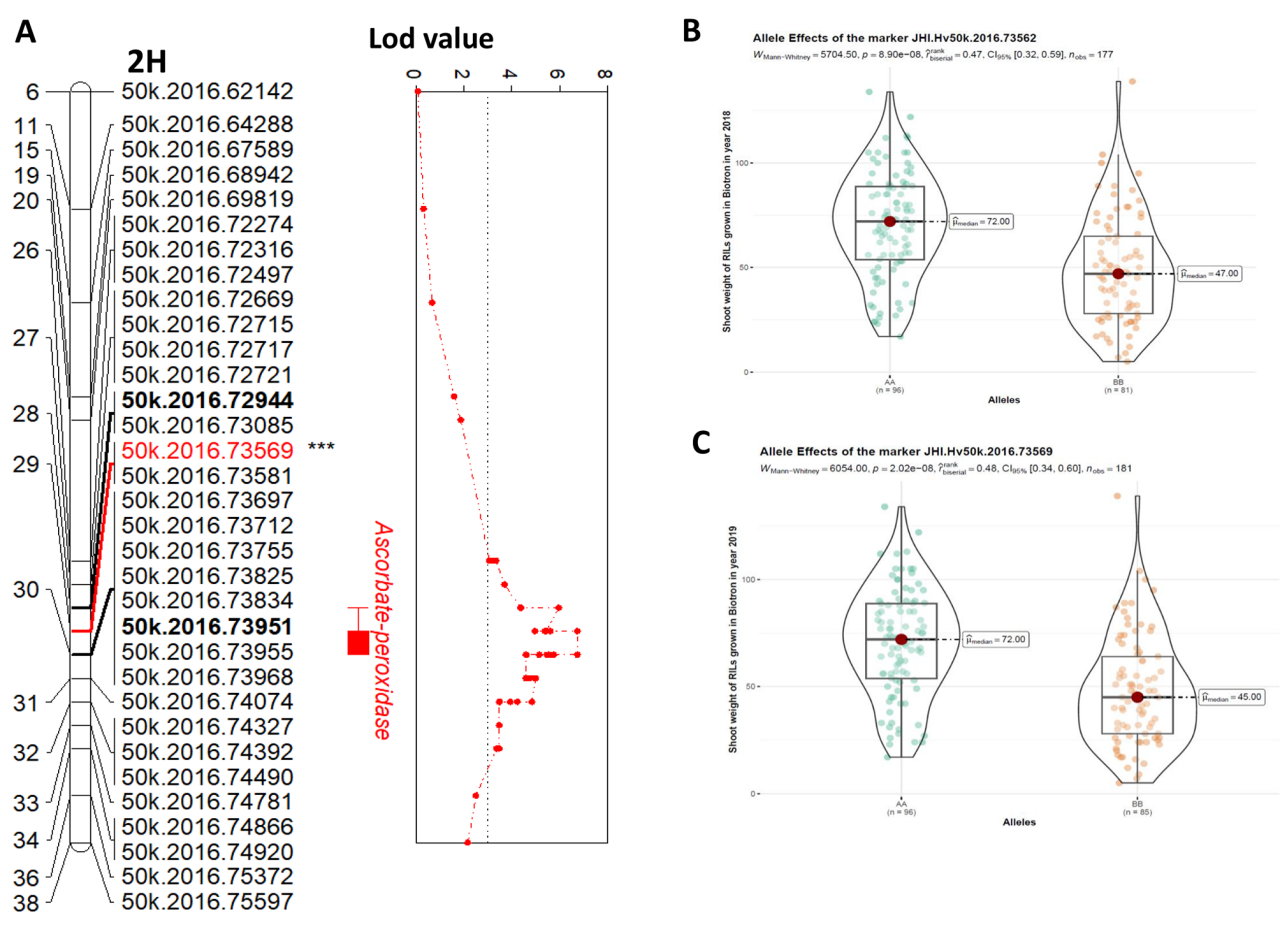
QTL associated with shoot weight and associated marker effects. **A.** QTL on chromosome 2 H with the peak LOD associated close to the APX gene. The qShW2H.1 and qShW2H.2 located at approximately 29.3 Mbp is closest to the ascorbate peroxidase gene which impacts abiotic stress such as drought. Interestingly, the marker 50k.2016.73569 corresponding to qShW2H.2 at 29.3 Mbp is located 850 bp upstream of the Ascorbate peroxidase gene and 57kbp from *Ppd-H1* gene which regulates barley flowering time during photoperiod response. Markers indicated in black bold corresponded to 1 LOD drop from the most significant QTL (indicated in red). **B, C.** Effect size plots of markers associated with shoot weight on chromosome 2 H. Effect size plot for markers JHI.Hv50k.2016.73562 (B) and JHI.Hv50k.2016.73569 (C)

Eight QTLs were detected on chromosome 2 H, 3 H, 4 and 5 H for shoot dry weight for biotron 2018 and 2019 (Additional Fig. 1D, E) of which three were on chromosome 5, two each on chromosomes 2 and 3, and one on chromosome 4. QTL on chromosome 4 and 5 H increased the phenotypic value for shoot dry weight in Otis, whereas QTL on chromosome 2 and 3 H contributed the alleles for higher shoot weight in GP (Table 3). Interestingly, *qShW2H.1* had the largest effect with the highest LOD scores (6.75) and explained 24.13% of the phenotypic variance for shoot dry weight for biotron 2018. The nearest marker QTL (JHI-Hv50k-2016-73569) was located around 850 bp upstream of the L-ascorbate peroxidase (*APX*) gene (*HORVU.MOREX. r3.2HG107740*) (Fig. 4A), and 57, 071 bp from the *Ppd-H1* gene which regulates photoperiod response and flowering time in barley. In the same genomic region, *qShW2H.2* was detected in biotron 2019 with LOD score of 4.78 and explained 18.19% of the phenotypic variance. Both QTLs on chromosome 2 H were above the genome-wide significance LOD threshold level and the GP allele at both loci increased shoot dry weight under drought stress (Fig. 4B C). The *qShW5H.2* and *qShW5H.3* were detected in biotron 2018 and 2019 and explained 7.47% and 17.57% of the phenotypic variation for shoot dry weight and the Otis allele at both loci increased shoot dry weight under drought stress (Table 3).

Seeds from each RIL collected in biotron in 2018 and 2019 were combined (referred to as ‘combined samples’ henceforth) due to a reduction in the number of seeds in the drought stressed plants, and fewer plants per RIL in the biotron. Four QTL were detected, two each on chromosomes 2H and 5H. The *qP2H.1* and *qP2H.2* were consistently detected in the same genomic region for combined samples and 2020 Idaho samples and explained 4.68% and 6.63% of the phenotypic variation for protein content. GP allele at *qP2H.1*, *qP2H.2* and *qP5H.1* contributed to the increased protein content under drought stress. Notably, *qP5H.2* detected in Idaho 2020 had the largest effect for protein content and explained 10.98% of the phenotypic variance and was contributed by the Otis allele.

Interestingly, *qP5H.2* was near *qShW5H.2* and *qShW5H.3* at the telomeric end of chromosome 5 H (Fig. 3). Also, the *qP2H.2* was 0.84 Mbp from the *qShW2H.2* influencing shoot dry weight. Given the stable QTLs observed on chromosome 2 H at approximately 28–30 Mbp and on 5 H at approximately 487–488 Mbp, candidate genes found within the vicinity of these QTLs were analyzed to determine their involvement in shoot weight, protein content and/or abiotic stress through literature search. A total of 62 genes were identified on 2 H between 28 and 30 Mbp, and 21 genes were identified on 5 H between 487 and 488 Mbp (Additional Table 2).

### Malt quality of drought-tolerant RILs

Based on an arbitrary cutoff of less than 20% reduction in seed yields compared to controls, five RILs were selected based on their consistent performance across the environments for malt quality analysis. Seeds from the drought stressed plants of these tolerant lines exhibited significant differences in their malt quality profiles (Table 4). For four of the traits (Diastatic power, Alpha amylase, % malt extract, and ratio of soluble to total protein) values were lower than that observed for GP under water stress. Furthermore, the beta-glucan content of all the five lines were 2-4-fold higher compared to GP. The RIL# 83 exhibited higher FAN and %wort soluble protein compared to GP but was also accompanied with higher beta-glucan and protein content that negatively impact malt quality.

**Table 4:** Means and standard deviation of malt quality parameters for high performing RIL, Otis and Golden Promise cultivars for year 2020 under dryland conditions

RIL	DP	AA	ME (%)	BG (ppm)	FAN (ppm)	Wort SP (%)	Total Protein (%)	S/T (%)
15	84 ± 6	37 ± 1	73 ± 1	1058 ± 94	141 ± 1	3.2 ± 0.04	13.34 ± 0.03	23 ± 0.4
23	93 ± 20	27 ± 6	72 ± 5	1039 ± 161	121 ± 3	3.11 ± 0.05	14.07 ± 0.41	21 ± 0.3
83	105 ± 8	51 ± 1	75 ± 1	744 ± 369	171 ± 1	4.0 ± 0.00	14.04 ± 0.11	27 ± 0.1
126	118 ± 1	25 ± 4	70 ± 2	1278 ± 8	98 ± 1	2.96 ± 0.06	15.98 ± 0.35	18 ± 0.1
150	71 ± 4	25 ± 1	72 ± 1	1309 ± 41	117 ± 4	3.17 ± 0.17	16.36 ± 0.88	19 ± 2.0
GP	124 ± 5	66 ± 2	78 ± 1	284 ± 144	154 ± 7	3.98 ± 0.13	13.74 ± 0.23	28 ± 0.5
Otis	82 ± 4	33 ± 2	74 ± 1	1248 ± 4	144 ± 18	3.38 ± 0.35	15.38 ± 0.59	21 ± 1.3

High performing lines were selected as described in materials and methods. AA, alpha amylase; DP, diastatic power; ME, Malt extract; BG, beta glucan; FAN, free amino nitrogen; SP, malt soluble protein; S/T, soluble protein/total protein; DWB, reported on dry weight basis

## Discussion

Drought, being the most damaging of all abiotic stressors, remains a formidable challenge for sustainable food production, as they can reduce yield by nearly 70% in crop plants [3]. The increased demand of barley for food, beer, and feed with concomitant reductions in yields perpetuated by climate change have accelerated research efforts towards understanding the genetic basis of drought associated agronomic traits and their responses in target environments. This approach enabled identification of QTL not only linked with yield, but also, physiological and biochemical responses to water deprivation [38]. Several abiotic stress tolerance mapping studies in barley have been reported [39, 40]. However, in these studies only few hundred markers were used for MTA and hence many highly informative QTL inherent in the genome remain uncharacterized. In this study, we used barley 50k iSelect SNP Array [41] which has been widely used [42–44].

Otis is a feed barley suited for the US-western drylands (with high temperatures and low moisture) and GP, a good malting variety, but sensitive to heat and drought stresses. The biparental population exhibited a spectrum of differences in their phenotypes, a manifestation of the wide differences in the parental lines, that enabled uncovering genetic footprints relevant to drought tolerance in barley. The broad and continuous range of variation in seed weight, shoot weight, and protein content measurements for the RIL population indicate these traits are controlled by QTL with additive effects.

The seed protein content from 2021 field harvest were significantly higher for both irrigated and rainfed conditions suggesting that these plants experienced some severe weather conditions. In fact, in the 2021 field year, significantly above average temperatures were recorded that was further exacerbated with a significantly lower than average rainfall during the tillering and heading stages (Additional Table 1). In our previous studies we have shown that a combination of heat and drought stress is detrimental to barley seed yields as well as its malting quality [37, 45, 46]. These climate factors could have contributed to the skewed protein levels in the 2021 field experiment. Hence the data from 2021 field grow out was not considered for further analysis.

Shoot characteristics play an important role in grain weight under terminal stresses such as drought and heat [29]. In barley, drought imposed at heading [46] and post-anthesis impairs photosynthesis, and impacts final grain weight, which decreases yield [47]. Under drought stress, we observed that seed weight of Otis cultivar was much higher than GP in all the environments investigated, a finding that is consistent with earlier report [37]. In addition, GP had higher shoot weight, while Otis had a lower shoot weight. These observations suggest limitations for efficient remobilization and translocation of assimilates to the developing seed under drought in GP. It is speculated that the nutrient reserves from stem that ought to be remobilized and translocated during seed filling, remain trapped in the stem [37] leading to higher shoot biomass and lower seed weights in drought sensitive GP. On the same lines, the lower shoot biomass in the tolerant Otis suggests efficient remobilization of the reserves that ensures adequate seed filling leading to higher seed weights. The seed filling processes in the developing and maturing caryopses are highly sensitive to environmental changes and influences the quality and quantity of the yield [48].

In a previous study we reported extensive differences in the transcriptome of Otis and GP under heat, drought and combined heat and drought stress [37]. Here, we sought to investigate the underlying drought tolerance QTL in the biparental population derived from Otis and GP. We detected 23 significant QTL (11 QTL for seed weight, 8 QTL for shoot weight and 4 QTL for protein content) in the GP/Otis mapping population. For seed weight trait, we observed that *qSW2H.1* and *qSW2H.2* located on chromosome 2 H were detected in greenhouse 2019 and field grown 2020 environments respectively, while four QTL on chromosome 5 H (*qSW5H.1*, *qSW5H.2*, *qSW5H.3* and *qSW5H.4*) were detected across both environments and years investigated. The remaining QTL associated with seed weight were only detected in single environment and found distributed across the chromosomes (except chromosome 1 and 3 H). All the QTL associated with seed weight in water deficit conditions were contributed by the Otis allele (except *qSW7H.2)*, which might explain the increase in seed weight detected in Otis compared to GP as observed in this study. This finding is consistent with an earlier report that observed a significant reduction in seed length and width in GP compared to Otis variety under drought stress due to alterations in the synthesis and distribution of carbohydrates or changes in cell wall properties [37].

Numerous QTL studies in bi-parental mapping have been conducted to identify genomic components conferring drought stress tolerance in barley [14, 32, 49–51]. Direct comparisons of our QTL mapping with those studies are complicated, given the different genotyping technologies and different linkage maps used in previous studies. However, we were able to validate our markers based on whether QTL were identified for the same or related traits at approximately congruous genomic positions. In our study, we observed that several QTL on chromosome 2 and 5 H were co-localized in the same region for shoot weight and protein content and were consistently detected across environments and may be considered stable QTL. Interestingly, for shoot weight, *qShW2H.1* (JHI-Hv50k-2016-73562) and *qShW2H.2* (JHI-Hv50k-2016-73569) are both located in the same genomic region corresponding to approximately 29.3 Mbp and this genomic region is nearest to a flanking marker (JHI-Hv50k-2016-73570) that has been previously associated with above ground biomass, number of grains per plant, kernel weight per plant and plant height [10]. In a previous work, marker BOPA2_12_30872 located at 29 Mbp on 2 H was detected in two different populations, TX9425 X Naso Nijo [52] and YSM1 X Gairdner [53] and was associated with water logging score trait in the biparental populations. In another barley study using double haploid lines from a cross between TX9425 (a Chinese landrace variety with superior drought and salinity tolerance) and a sensitive variety, Franklin, this genomic region (29 Mbp) colocalized with the QTL associated with relative moisture content [14]. The presence of QTL conditioning different traits on a chromosome are indicative of the presence major gene(s) impacting multiple traits (pleiotropic effects). Such linked genes (traits) can vastly enhance selection efficiency when incorporated into marker assisted breeding. These observations lend credence to the fact that this genomic location has a pleiotropic effect on multiple traits which prompted us to identify candidate genes within this region, especially when markers within the QTL had high LOD scores that were greater than six with significant effect sizes (Fig. 4B and C). Markers JHI-Hv50k-2016-73566 and marker JHI-Hv50k-2016-73569 were approximately 3465 bp and 800 bp upstream of the ascorbate peroxidase (*APX*) gene that is well known to function in abiotic stress tolerance [54]. APX belong to type I heme peroxidase and copper oxidase family and rapidly scavenge hydrogen peroxide (H_2_O_2_) in ascorbic acid and glutathione cycle [55]. With *qShW2H.2* (JHI-Hv50k-2016-73569) having the largest effect QTL favoring GP allele for shoot dry weight (Fig. 4C), it is tempting to speculate that *APX* gene may play a pivotal role in drought tolerance, but additional work is needed to verify this hypothesis. One of the 62 candidate genes identified (Additional Table 2), is the photoperiod response gene, *Ppd-H1* gene, which is well characterized and known to regulate barley flowering time through photoperiod response [56]. The *Ppd-H1* gene was approximately 57 kbp from marker JHI-Hv50k-2016-73566 nearest to the *APX* gene. It has been reported that *Ppd-H1* gene interacts with drought stress signals to modulate spike development in barley [57]. It would be interesting to verify the precise role of *Ppd-H1* and *APX* genes with respect to drought tolerance in barley.

Because chromosome 5 H at approximately 487 Mbp genomic region is consistently detected across biotron experiments for shoot weight trait and seed protein content for field studies, we further investigated the candidate genes within this region to assess their functional roles in drought tolerance. Twenty-one genes were identified within a 2 Mbp window, of which 13 genes have confirmed roles in drought tolerance based on literature search (Additional Table 2). Interestingly, marker JHI-Hv50k-2016-308239 contained a *c.733G > A* SNP site that is located within the *Dirigent* (*DIR*) gene (HORVU.MOREX. r3.5HG0480940), resulting in a missense mutation *p.Gly245Ser* on the fourth exon. We speculate this SNP may have functional significance since it is also annotated (vcZ0Y7P7) as a variant in the Barley MorexV3_pseudomeolecules_assembly genome. *DIR* genes have been widely implicated in drought, salt and oxidative stresses [58] and the expression of the most responsive *DIR* genes has been demonstrated to be correlated with increased lignification upon exposure to abiotic stress [59]. Further functional studies are warranted to examine the *p.Gly245Ser* missense mutation and its impact on *DIR* protein function during drought.

We observed that *qP5H.2* (JHI-Hv50k-2016-308379) contained an intronic G > A SNP site that is located within PDZ_6 domain-containing gene (HORVU.MOREX. r3.5HG0481070), while *qShW5H.3* (JHI-Hv50k-2016-308419) is in the intergenic regions of two PDZ_6 domain-containing genes (HORVU.MOREX. r3.5HG0481080 and HORVU.MOREX. r3.5HG0481090). PDZ-domain containing proteins are membrane-associated proteins that have been previously linked with detoxification of reactive oxygen species resulting from salt stress [60] and the redox network of the chloroplast [61]. While the marker JHI-Hv50k-2016-308379 is annotated (vcZ2300N2) as a variant in the Barley MorexV3_pseudomeolecules_assembly genome, the other marker JHI-Hv50k-2016-308419, sandwiched between two PDZ_6 domain-containing genes (HORVU.MOREX. r3.5HG0481080 and HORVU.MOREX. r3.5HG0481090) appeared to be novel. We speculate that these markers (JHI-Hv50k-2016-308379 and JHI-Hv50k-2016-308419) nestled among PDZ_6 domain-containing genes influences transcriptional responses of these genes and other nearby candidate genes during stress events in barley and warrants further investigation (Additional Table 2).

Drought stress does not only contribute to reduced grain weight but also leads to an increase in total protein content [62] which is a negative attribute for a malting variety. Increased protein content in Otis compared to GP is not surprising since the former is a feed variety. The two QTL, *qP2H.1* and *qP2H.2*, corresponding to marker JHI-Hv50k-2016-73085 and JHI-Hv50k-2016-73825 respectively, were associated with protein content on chromosome 2 H, and reside within the same genomic region identified for shoot dry weight. Although, different QTL regions (*qP5H.1* and *qP5H.2*) on chromosome 5 H were associated with protein content, the *qP5H.2* appears to be pleiotropic since it is in the same genomic region as QTL influencing shoot dry weight and could plausibly explain the underlying connections between nitrogen storage reserves in the leaves, stored nitrogen in plant cells, and protein content during grain filling under drought conditions [63]. Notably, the average value of the protein content in the RIL population (> 16.5%) is higher than the acceptable values for malting (11.8–12.8%) (www.ambainc.org). Earlier works showed that high protein content reduced both the malt extract and Kolbach index but positively correlated with diastatic power [64] while abiotic stress such as drought increases barley protein content [65]. The five RILs selected based on their lower seed yield loss under drought, exhibited a malt quality profile that was overall poorer compared to GP. Most notable was the high beta glucan content that has been reported to increase viscosity of wort and cause difficulty in filtering and chill haze in bright beer [66].

## Conclusions

The GP X Otis RIL population is a valuable resource for identifying QTL associated with stress tolerance in barley. Consistent with a previous meta-analysis of QTL associated with abiotic stresses [67], Chromosome 2 and 5 H contained QTL that impacted shoot weight and seed traits in response to drought stress during heading stage. The pivotal role of reactive oxygen species in stress signaling supports further functional analysis of the *AP*X gene on chromosome 2 H and the *DIR* gene on chromosome 5 H identified in this study. Furthermore, along with these two candidate genes the PDZ6-domain genes can also be utilized in marker-assisted selection programs for improving drought tolerance in barley. Since both drought and malting quality are complex traits, identifying the RILs with genetic network rewiring necessary for drought adaptive responses of Otis along with favorable malting quality attributes of GP may be realized by screening a larger RIL population.

## Materials and methods

### Mapping population

The RIL mapping population was developed from a cross between Golden Promise (GP), a popular spring malting barley variety (a gamma-ray mutant of the Scottish cultivar Maythorpe) and ‘Otis’, a high-yielding US spring feed barley variety developed in the 1960s especially for the dryland conditions [68]. The F_1_ plants were selfed and advanced by single seed descent in Aberdeen Idaho, United States for five generations. Of the approximately 220 F_1_ lines, 192 lines survived over the years and was used for this analysis. Previous study by our group reported that Otis was tolerant to drought stress compared with GP based on seed yield [37]. Along with the seed yields, in this study the seed protein content as well as the shoot weight in response to drought were evaluated for the RIL population.

### Biotron experiments

The soil mix for this study consisted of vermiculite, peat moss and sand (1:1:2). The three components were blended in a concrete mixer along with 25 g of slow release 15-9-12 osmocote fertilizer (Scotts) per kg of mix. Pots were filled with the mix to a pre-determined weight to ensure consistent amount of potting mix in each of the pots. Seeds of GP, Otis, and 192 RILs were imbibed with water for three hours and three seeds were sown in each 2.5 L pots in a randomized complete block design in the biotron facility, at the University of Wisconsin, Madison. Plants were grown with 16-hour light (400 µmol m^− 2^ s^− 1^) at 22 ^o^C and eight hours of darkness at 18 ^o^C. Relative humidity in the biotron was around 50%. For each RIL, four pots were maintained for pursuing drought treatment and four pots for well-watered controls. On each table there were at least two pots of the two parental lines as checks. Plants were set to auto-irrigation about three weeks after emergence. Drought stress was imposed by withholding irrigation prior to the full emergence of the heads from the main tiller corresponding to Zadok’scale: 5.9. Plants were subjected to progressive drought for a period of five days. Soil moisture was monitored every day using soil moisture probe and by the end of the fifth day after withholding irrigation, the soil moisture in the drought pots were between 10 and 20% compared to the well-watered control pots that were maintained at about 60% of field capacity on weight basis. After five days of progressive drought, the pots were set for auto-irrigation till the end of physiological maturity. Heads were harvested from each plant separately and threshed using a bench top thresher (Model LT15, Haldrup, Poneto, IN). Seed weight per plant was recorded. Dry shoot biomass was collected for each plant separately. Biotron studies were conducted between March and July in 2018 and 2019.

### Field experiments

In the years 2020 and 2021, field experiments were conducted with the parental lines GP, Otis and the 192 RILs in Aberdeen, Idaho, under irrigated and rainfed conditions. Seeds were planted in 1.5 m long rows and the seeding rate was 5 g per row. Three head rows were planted in the dryland conditions and two were maintained under irrigated conditions for each RIL. For the years 2020 and 2021, planting dates were between April 6th-12th, heading dates were between June 20th-30th and the harvest dates were August 5th-15th. The soil type in this field was DeA Declo Loam. Fertilizer management for the field comprised total N at 255 Kg/Hectare and total P at 233 Kg/Hectare in 0–60-centimeter soil depth. Water application rates were about 8 h/week and for the irrigated plots amounted to 10 cm in May, 90 cm across the month of June, and 35 cm in two weeks of July. For the rainfed plots the irrigation was limited to 10 cm in May and 6 cm during June. The heads from each row for each RIL in the field were threshed in Aberdeen and the total seed weight per row were recorded. The daily average temperatures and the amount of rainfall for the field location were collected for the months of April through August (Additional Table 1) (https://www.wunderground.com/history/monthly/us/id/pocatello/KPIH/).

### Traits evaluated

Data collected were seed weight (SW) from the four environments (biotron 2018, biotron 2019 (average seed weight/ plant) and field evaluations in Aberdeen, ID in 2020 and 2021 (seed weight/row); shoot dry weight (ShW is the average shoot weight/plant) of plants grown in biotron 2018 and biotron 2019, and seed protein content (P) that were estimated using FOSS NIR Infratech™ NOVA. Harvested seeds from 2018 to 2019 biotron were combined for the assessment of the protein content.

### Statistical analysis

Broad sense heritability estimates for each trait were estimated by considering the genotypes, location, year, and their interactions. Population statistics and analysis of variance (ANOVA) in individual environments was conducted in *R* version 4.2.1, while correlation between traits was analyzed using the R *corrplot* package, where the line mean for each trait in each environment was used as the input.

### Single nucleotide polymorphism (SNP) genotyping and linkage map construction

Four-week-old leaf samples from the parent lines and 192 F_5:6_ RILs were collected for DNA extraction and genotyping was done with the barley 50k iSelect SNP Array (Illumina Inc., San Diego, CA, USA) [69]. SNP data were processed using Genome Studio software. Data was filtered to remove monomorphic markers, and markers with greater than 60% missing data. After filtering steps followed by the removal of co-segregating markers, the remaining markers were used in linkage map construction using the R/qtl package [70] and genetic distance between markers was computed using Kosambi’s mapping function [71].

### QTL mapping

The mean trait value for each RIL within environment was used as input for the QTL analysis. Following the estimation of genotypic probabilities using the Hidden Markov Model (HMM), the maximum likelihood via the Expectation Maximization (EM) algorithm [72] was used to identify significant QTL. A threshold of log of odds (LOD) ≥ 2.0 was used to declare suggestive QTL [73]. Also, a total of 1000 genome-wide permutations were used to calculate the significant LOD threshold. QTLs were assumed to be different if their confidence intervals did not overlap. A significant QTL was considered stable if a genomic marker associated with the QTL consistently appeared in multiple traits or environments. QTL were designated by ‘q’ representing qtl, followed by trait (SW, Seed weight; ShW, shoot weight; P, NIR protein content of seeds), chromosome number, and serial number of the qtl on the chromsome. The significant markers identified in this study were compared with known QTLs from GrainGenes database (http://www.graingenes.org/) and literature. In addition, the GrainGenes database was used to search for additional marker and chromosomal information which was used for candidate gene analysis in barley. Visualization of QTL locations on barley chromosomes was performed using MapChart 2.32 [74].

### Candidate gene identification

Using Barleymap Morex_v3 assembly (*Hordeum_vulgare* - Ensembl Genomes 55), candidate gene identification was conducted by investigating the genes in a pre-defined flanking window of 1 Mbp upstream and down-stream of the stable QTL. The predicted genes were examined for their role with the associated phenotypes and their significance in the context of abiotic stress, especially drought tolerance using published literature.

### Malt quality evaluation

The seed yields per plant (drought-treated and corresponding well-watered controls) from the biotron experiments and the seed yields per row (rainfed versus irrigated) were ranked to identify top performing RILs. An arbitrarily chosen 20% yield loss was considered as the cutoff for selecting high performing lines. Five RILs (RIL lines: 15, 23, 83, 126, and 150) that performed well consistently across the years in the biotron experiments and the 2020 field study were selected. Seeds (2 g per replicate) from the rainfed field samples were micro-malted at the Cereal Crops Research Unit Quality lab (Madison, WI) using the methods described earlier [75]. Diastatic Power (DP), Alpha Amylase (AA), Malt Extract (ME), Beta Glucan (BG), Free Amino Nitrogen (FAN), Malt Soluble Protein (SP) and Soluble Protein to Total Protein ratio (S/T) were analyzed to assess malt quality profile using methods and procedures recommended by the American Society of Brewing Chemists as described [76]. The malt quality metrics of these select lines were compared to Otis and GP.

## Electronic supplementary material

Below is the link to the electronic supplementary material.

Supplementary Material 1

## Data Availability

The genotyping and phenotyping data for the RIL population will be made available through the Triticeae T3 toolbox (https://barley.triticeaetoolbox.org/). The seeds of the RIL mapping population is deposited at the National Laboratory for Genetic Resources Preservation, Fort Collins, Colorado.

## List of abbreviations

AA: Alpha Amylase
APX: Ascorbate Peroxidase
BG: Beta Glucan
DP: Diastatic Power
FAN: Free Amino Nitrogen
GP: Golden Promise
Mbp: Million basepairs
ME: Malt Extract
qP: Quantitative trait loci associated with seed protein content
qSh: Quantitative trait loci associated with shoot weight
qSW: Quantitative trait loci associated with seed weight
QTL: Quantitative Trait Loci
RIL: Recombinant Inbred Line
SNP: Single Nucleotide Polymorphism
SP: Soluble Protein
S/T: Soluble Protein to Total Protein ratio
