# Supplementary material for: QTL mapping of shoot and seed traits impacted by Drought in Barley using a recombinant inbred line Population
Source: BMC Plant Biol. 2023 May 27;23:283. doi: 10.1186/s12870-023-04292-x (PMC10224580; doi:10.1186/s12870-023-04292-x)
Supplement: Supplementary file 1 — Supplementary Material 1 [file 12870_2023_4292_MOESM1_ESM.pdf]

Additional Table 1. Temperature and rainfall data for the Aberdeen field studies in 2020 and 2021																
		April			May			June			July			August		
Temp. (ºF)	Year	Max	Avg	Min	Max	Avg	Min	Max	Avg	Min	Max	Avg	Min	Max	Avg	Min
	2020	61.58	46.39	28.39	74.32	56.03	40.49	77.75	62.66	44.74	81.90	71.38	63.11	82.38	73.33	54.58
	2021	68.04	45.53	34.37	65.21	55.39	44.38	80.56	71.98	55.43	82.32	76.43	55.17	81.25	67.44	45.97
Rainfall (inches)		Max	Avg	Min	Max	Avg	Min	Max	Avg	Min	Max	Avg	Min	Max	Avg	Min
	2020	0.63	0.05	1.40	0.62	0.04	1.39	0.47	0.06	1.72	0.11	0.01	0.21	0.10	0.00	0.11
	2021	0.38	0.04	1.16	0.32	0.02	0.57	0.01	0.00	0.01	0.01	0.00	0.01	1.03	0.05	1.47

Additional Table 2. Candidate gene list within 1Mbp upstream and downstream of stable QTL on chromosome 2H and 5H.

ID ofcandidate genes on chrom 2H	Uniprot name
**HORVU.MOREX.r3.2HG0107220**	Growth arrest and DNA damage-inducible protein
**eiHORVU.MOREX.r3.2HG0107250**	Glycine-rich cell wall structural protein 2-like
HORVU.MOREX.r3.2HG0107260	Str_synth domain-containing protein
HORVU.MOREX.r3.2HG0107280	DUF295 domain-containing protein
**HORVU.MOREX.r3.2HG0107300**	Acetyltransferase
**HORVU.MOREX.r3.2HG0107310**	NAM-associated domain-containing protein
HORVU.MOREX.r3.2HG0107320	DUF295 domain-containing protein
HORVU.MOREX.r3.2HG0107330	Coiled-coil domain-containing protein 167
HORVU.MOREX.r3.2HG0107340	PMR5N domain-containing protein
HORVU.MOREX.r3.2HG0107350	DUF295 domain-containing protein
**HORVU.MOREX.r3.2HG0107380**	Dof-type domain-containing protein
HORVU.MOREX.r3.2HG0107400	Protein kinase domain-containing protein
**HORVU.MOREX.r3.2HG0107410**	MATH domain-containing protein
HORVU.MOREX.r3.2HG0107420	Oxoglutarate dehydrogenase (succinyl-transferring)
**HORVU.MOREX.r3.2HG0107430**	HTH myb-type domain-containing protein
HORVU.MOREX.r3.2HG0107440	p53 and DNA damage-regulated protein 1
HORVU.MOREX.r3.2HG0107450	DUF4378 domain-containing protein
HORVU.MOREX.r3.2HG0107470	Pectate lyase
HORVU.MOREX.r3.2HG0107480	LITAF domain-containing protein
**HORVU.MOREX.r3.2HG0107490**	DUF569 domain-containing protein
**HORVU.MOREX.r3.2HG0107500**	DUF3444 domain-containing protein
**HORVU.MOREX.r3.2HG0107550**	DUF569 domain-containing protein
**HORVU.MOREX.r3.2HG0107570**	Bidirectional sugar transporter SWEET
HORVU.MOREX.r3.2HG0107590	NAB domain-containing protein
**HORVU.MOREX.r3.2HG0107600**	Expansin-B2
**HORVU.MOREX.r3.2HG0107610**	Expansin-B2
**HORVU.MOREX.r3.2HG0107620**	Glutamic acid-rich protein-like

**HORVU.MOREX.r3.2HG0107640**	Expansin-B11
**HORVU.MOREX.r3.2HG0107650**	Glycosyltransferase
**HORVU.MOREX.r3.2HG0107660**	Transposase
**HORVU.MOREX.r3.2HG0107680**	BHLH domain-containing protein
**HORVU.MOREX.r3.2HG0107690**	Serine/threonine kinase
HORVU.MOREX.r3.2HG0107700	Uncharacterized protein
**HORVU.MOREX.r3.2HG0107710**	PPD-H1
HORVU.MOREX.r3.2HG0107720	Str_synth domain-containing protein
HORVU.MOREX.r3.2HG0107730	DUF155 domain-containing protein
**HORVU.MOREX.r3.2HG0107740**	L-ascorbate peroxidase
HORVU.MOREX.r3.2HG0107750	Arogenate dehydratase
HORVU.MOREX.r3.2HG0107760	Reverse transcriptase Ty1/copia-type domain-containing protein
**HORVU.MOREX.r3.2HG0107770**	Myb_DNA-bind_3 domain-containing protein
HORVU.MOREX.r3.2HG0107780	TPD1 protein-like 1
HORVU.MOREX.r3.2HG0107790	HHH_2 domain-containing protein
HORVU.MOREX.r3.2HG0107800	PWWP domain-containing protein
**HORVU.MOREX.r3.2HG0107820**	Glycosyltransferases
**HORVU.MOREX.r3.2HG0107830**	Glycosyltransferases
HORVU.MOREX.r3.2HG0107850	Elongation factor 1-alpha
HORVU.MOREX.r3.2HG0107900	Elongation factor 1-alpha
HORVU.MOREX.r3.2HG0107910	Metal-nicotianamine transporter YSL6
**HORVU.MOREX.r3.2HG0107940**	MLO-like protein
**HORVU.MOREX.r3.2HG0107950**	zf-GRF domain-containing protein
**HORVU.MOREX.r3.2HG0107960**	Phosphoinositide phospholipase C
**HORVU.MOREX.r3.2HG0107970**	Peroxidase
HORVU.MOREX.r3.2HG0107980	DUF3453 domain-containing protein
**HORVU.MOREX.r3.2HG0107990**	Protein FAR1-RELATED SEQUENCE
**HORVU.MOREX.r3.2HG0108000**	Protein FAR1-RELATED SEQUENCE
HORVU.MOREX.r3.2HG0108010	Uncharacterized protein

HORVU.MOREX.r3.2HG0108020	Predicted protein
**HORVU.MOREX.r3.2HG0108040**	V-SNARE coiled-coil homology domain-containing protein
**HORVU.MOREX.r3.2HG0108050**	PHD-type domain-containing protein
HORVU.MOREX.r3.2HG0108060	Uncharacterized protein
HORVU.MOREX.r3.2HG0108080	FAD-binding PCMH-type domain-containing protein
**HORVU.MOREX.r3.2HG0108090**	Dirigent protein
Gene ID for candidate genes on chrom5H	
**HORVU.MOREX.r3.5HG0480850,**	Auxin-responsive protein SAUR50-like
**HORVU.MOREX.r3.5HG0480870,**	AAA domain-containing protein
**HORVU.MOREX.r3.5HG0480890,**	**Dirigent protein**
**HORVU.MOREX.r3.5HG0480940,**	**Dirigent protein**
HORVU.MOREX.r3.5HG0480960,	TMV resistance protein N-like
HORVU.MOREX.r3.5HG0481010,	Predicted protein
**HORVU.MOREX.r3.5HG0481030,**	Embryogenesis-associated protein EMB8
**HORVU.MOREX.r3.5HG0481070,**	**PDZ_6 domain-containing protein**
**HORVU.MOREX.r3.5HG0481080,**	**PDZ_6 domain-containing protein**
**HORVU.MOREX.r3.5HG0481090,**	**PDZ domain-containing protein**
**HORVU.MOREX.r3.5HG0481100,**	LEA_2 domain-containing protein
HORVU.MOREX.r3.5HG0481110,	RBR-type E3 ubiquitin transferase
**HORVU.MOREX.r3.5HG0481120,**	RING-type domain-containing protein
HORVU.MOREX.r3.5HG0481130,	Secreted protein
**HORVU.MOREX.r3.5HG0481140,**	F-box/kelch-repeat protein SKIP11
HORVU.MOREX.r3.5HG0481160,	Glycine--tRNA ligase
**HORVU.MOREX.r3.5HG0481240,**	AP2/ERF domain-containing protein
HORVU.MOREX.r3.5HG0481290,	Predicted protein
HORVU.MOREX.r3.5HG0481310,	ARID domain-containing protein
HORVU.MOREX.r3.5HG0481320,	Alanine transaminase
**HORVU.MOREX.r3.5HG0481330,**	GRF-type domain-containing protein

N.B Those indicated in bold are implicated in abiotic stress based on published literatures. Ascorbate peroxidase, PPD-H1 on 2H, and Dirigent and PDZ_6 domain-containing protein genes on 5H are nearest to the stable QTL and indicated in red.

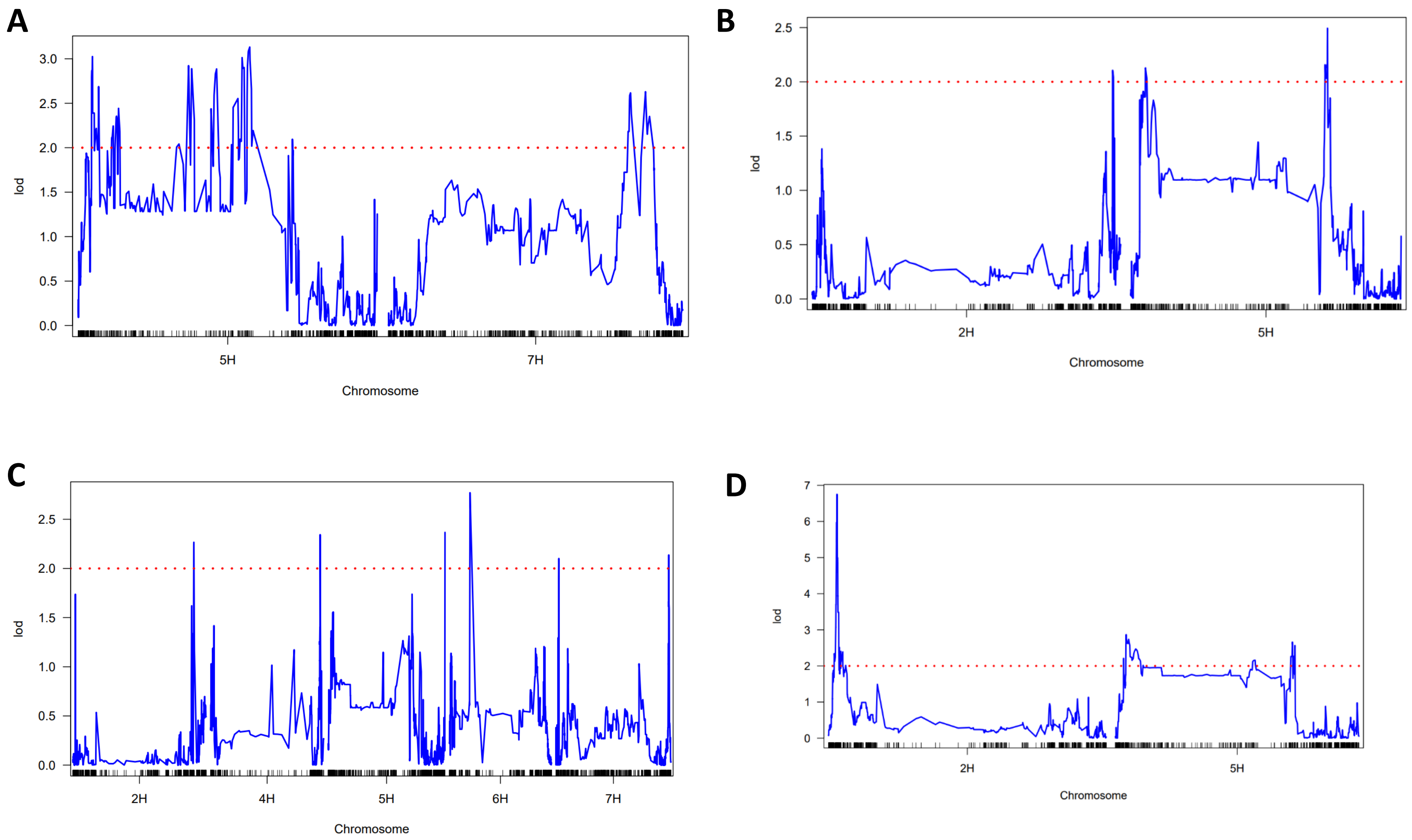

A
lod
0.0
0.5
1.0
1.5
2.0
2.5
3.0
5H
7H
Chromosome
B
lod
0.0
0.5
1.0
1.5
2.0
2.5
2H
5H
Chromosome
C
lod
0.0
0.5
1.0
1.5
2.0
2.5
2H
4H
5H
6H
7H
Chromosome
D
lod
0
1
2
3
4
5
6
7
2H
5H
Chromosome

E

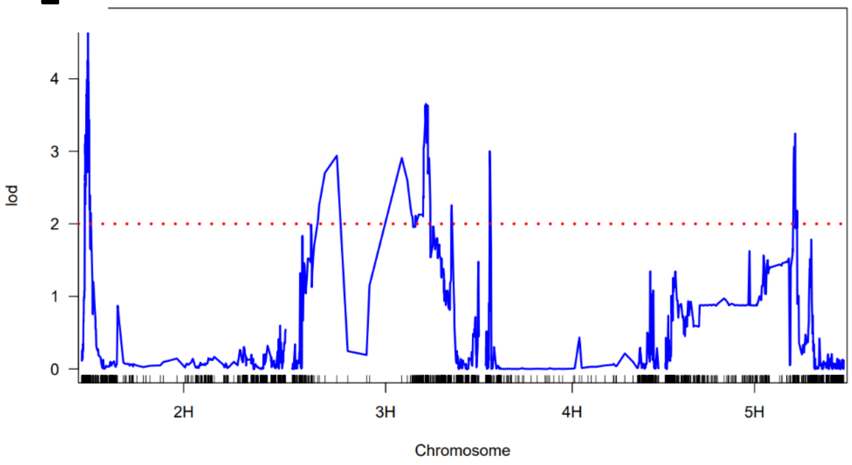

F

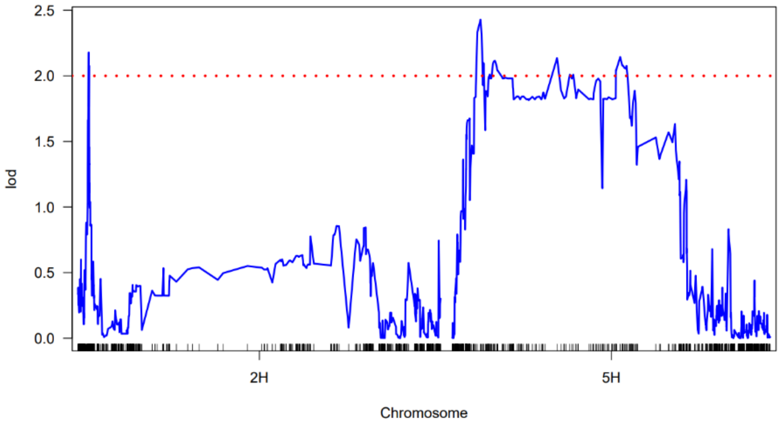

G

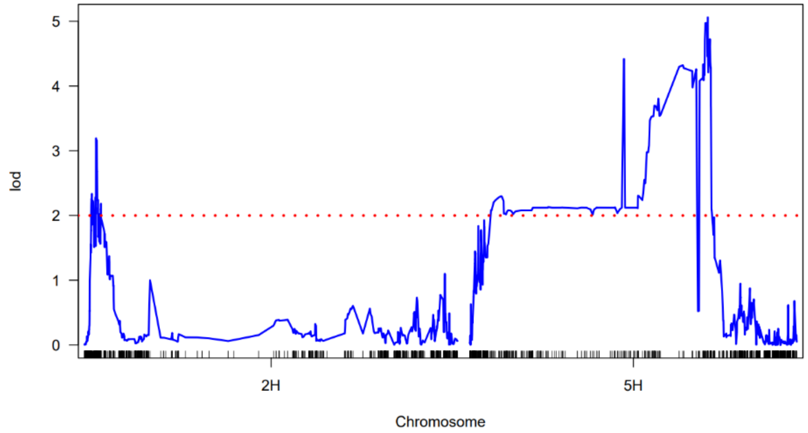

Additional figure 1. QTL plot of RIL population for (A) seed weight data for biotron 2018, (B) seed weight data for biotron 2019, (C) seed weight data for Idaho 2020, (D) Shoot dry weight for biotron 2018, (E) Shoot dry weight for biotron 2019, (F) Protein content for combined 2018 and 2019 seed samples, (G) Protein content for Seed samples from Idaho 2020.
